# Associations of neighborhood physical and crime environments with obesity-related outcomes in Jamaica

**DOI:** 10.1371/journal.pone.0249619

**Published:** 2021-04-05

**Authors:** Colette Cunningham-Myrie, Katherine P. Theall, Novie Younger-Coleman, Lisa-Gaye Greene, Parris Lyew-Ayee, Rainford Wilks

**Affiliations:** 1 Department of Community Health and Psychiatry, University of the West Indies, Mona, Jamaica; 2 Department of Global Community Health and Behavioral Sciences, School of Public Health and Tropical Medicine, Tulane University, New Orleans, LA, United States of America; 3 Caribbean Institute for Health Research, University of the West Indies, Mona, Jamaica; 4 Mona GeoInformatics Institute, University of the West Indies, Mona, Jamaica; Graduate School of Public Health and Health Policy, City University of New York, UNITED STATES

## Abstract

**Objective:**

To examine whether proximity and density of public open spaces, public parks, street connectivity, and serious and violent crimes were associated with Body Mass Index (BMI) and Waist Circumference (WC) within and across levels of urbanicity, sex and socioeconomic status (SES) in Jamaica, a small island developing state (SIDS).

**Methods:**

Secondary analysis was conducted using data from the Jamaica Health and Lifestyle Survey 2008 (JHLS II). All respondents were geocoded to area of residence in Enumeration Districts (EDs). Intraclass correlation coefficients (ICCs) were derived and multilevel mixed effects regression models applied to 2529 participants nested within 101 EDs from all 14 parishes in Jamaica.

**Results:**

There was significant clustering across neighborhoods for mean BMI (ICC = 4.16%) and mean WC (ICC = 4.42%). In fully adjusted models statistically significant associations included: increased mean BMI among men, with increased intersection density/ km^2^ (β = 0.02; 95% CI = 1.96 x10^-3^, 0.04, *p* = 0.032); increased mean WC among urban residents with increased crimes/km^2^/yr (β = 0.09; 95% CI = 0.03, 0.16, *p*<0.01) and among persons in the middle class, with further distance away from public parks (β = 0.30; 95% CI = 0.08, 0.53, *p*<0.01).

**Conclusions:**

Neighborhood physical and crime environments were associated with obesity-related outcomes in Jamaica. Policymakers in SIDS such as Jamaica should also note the important differences by urbanicity, sex and SES in prevention efforts designed to stem the growing obesity epidemic.

## Introduction

The worldwide prevalence of obesity has nearly doubled between 1980 and 2008 [1]. Obesity is now a common condition on every continent [2, 3] and is increasing in developing countries [4]. There remains co-existence of obesity with undernutrition within countries and within households creating a ‘double-burden’ of disease, which threatens to overwhelm health services [4] and increase health care expenditure [5] of low and middle-income countries [LMICs], many of which are also small island developing states [SIDS].

There are significant sex differences in the prevalence of obesity, with the condition posing a serious problem among women in Latin America, the Caribbean, Middle East, North Africa, the Central Eastern Europe = Commonwealth of Independent States (CEE = CIS) and the USA [6]. A dramatic rise in the rate of obesity has been observed in the Caribbean over the past four decades [7] including observations from the use of cross-cultural studies from Jamaica, St. Lucia and Barbados [8]. Additionally, in contrast with other geographic areas, the data reveal in many instances, higher prevalence rates among Caribbean women [9, 10].

Specifically, in Jamaica data from the two Jamaica Health and Lifestyle Surveys (JHLSs) completed in 2000 (JHLS I) and 2008 (JHLS II) among 15-74-year-old persons revealed an increase in prevalence of overall obesity (19.7% versus 25.3%) and significant sexual dimorphism for both surveys [females: 29.9% vs. 37.7%; males: 9.6% vs. 12.4%] [9]. The JHLS II [9] also revealed that the prevalence of increased WC was significantly higher among females versus males (69.8% vs. 19.6%; p<0.001). Another study, the Jamaica Youth Risk and Resiliency Survey [10] revealed that 6% of 15–19-year-old adolescents were obese, with significantly higher prevalence of obesity in females (females: 8% vs. males: 3.3%).

Lifestyle behaviors that drive obesity, including physical inactivity and low consumption of fruit and vegetables, have continued to increase [9], despite many national and local health promotion programs targeting individual-level prevention [11, 12]. There is now the need to focus on the extent of the contribution of more distal social and community factors driving this epidemic, which hitherto have not been investigated in Jamaica. Many of these community exposures are related to socioeconomic status (SES), which has been linked to adiposity.

The role of SES on measures of adiposity has also been examined in countries at different stages of the epidemiological transition with some inconsistencies. For example, in a systematic review published on SES and obesity studies conducted in adult populations from developing countries between 1989 and 2003, the burden of obesity in a particular developing country tended to shift towards persons of lower SES as the country’s Gross National Product increased [13]. On the other hand, in the Modeling the Epidemiologic Transition Study (METS), with about 500 participants aged 25–45 years in each of five sites (Ghana, South Africa, Jamaica, Seychelles, United States), obesity was more prevalent among individuals in the middle wealth tertile (based on household’s ownership of a number of assets) in both South African and Jamaican populations, but not so in the USA [14]. In the same study, an inverse gradient was observed (higher obesity rates with increasing wealth) for the Ghanian.

Literature out of North America [15], Asia [16, 17], Africa [18] and Europe [19] reveal that obesity-related outcomes and environmental influences may also differ by degree of urbanicity, with inconsistencies in the directionality of the associations across and within countries. Studies in Jamaica have in general assessed geographical variations in health outcomes on obesity according to either of the dichotomized classifications of urban or rural area of residence [20, 21]. Furthermore, there is a dearth of studies that have examined urban-rural differences in obesity-related outcomes. Results of the primary analysis of the JHLS II revealed a higher prevalence in urban areas for both mean BMI [urban: 26.5% versus rural 27.0%] and increased WC [urban: 46.0% versus rural: 43.7%] [4].

Geographic variations of obesity prevalence and trends may be linked to community context. There are several possible casual pathways to obesity that link body mass index (BMI), a measure of adiposity, to physical (natural and built) environments that influence physical activity (PA) and social environments such as safety and violence [22, 23]. Multiple interrelationships exist across domains as well as the presence of reciprocal pathways [22]. Key community contexts that have been examined in relation to obesity-related outcomes include proximity to and availability of open spaces such as public parks and community connectivity. Studies conducted in Australia found that individuals living within close proximity to public open spaces achieved higher levels of recreational walking [24] especially when these spaces were large and attractive [25, 26]. However not all research findings have been consistent in terms of the direction of this association [27]. Community connectivity, or the number and directness of transportation linkages between destinations [28], has also been linked to obesity. Specifically, lower street connectivity necessitates the use of automobiles for transport and is associated with an increased risk of obesity [29–31]. Some studies in the United States have shown that more connected street networks can promote physical activity, especially walking [32], and have been linked to decreased weight and waist circumference [WC] [33].

Beyond the physical environment, the social environment also may play a significant role in obesity rates and possibly by sex, given a hypothesized increased susceptibility and/or exposure of women to neighborhood effects [34]. Crime and perceived threats to safety have been associated with the ability to engage in PA and its subsequent effect on adiposity. A systematic review published in 2016 by Yu et al [35] suggest that relationship between crime and obesity operate through both individual and community-level mechanisms. At the individual level perception of safety may serve as a barrier to participation in outdoor PA-related activities and at the community level crime could operate indirectly on physical activity by fueling neighborhood decline. Epidemiologic studies have corroborated these hypotheses [36–39].

The limited body of research on environment influences on the Chronic Non-Communicable Diseases (CNCDs) in Jamaica and the developing world, as well as the apparent lack of lifestyle changes despite many health promotion programs targeting individual-level prevention, suggests that barriers to these changes may yet be unrecognized and accounted for in the traditional modeling of risk factors. The purpose of this study was to examine the roles of the physical and crime neighborhood environments in shaping obesity risk in Jamaica, providing a unique and important opportunity that addresses these gaps in understanding the environmental mechanisms influencing obesity, key risk factors and comorbidities in this context.

Specifically, the aim of this study was to explore whether neighborhood physical and crime environments were associated with both outcomes of mean BMI and mean WC within and across levels of urbanicity, sex and socioeconomic status (SES) in Jamaica. We hypothesized that a) the pathway between factors such as less open spaces (including public parks), less street connectivity, less accessibility/further proximity to open spaces, mediated through physical activity level (PAL), would lead to higher measures of adiposity and would be stronger for those of low SES and for women, and b) the pathway between crime density, mediated through low PAL, will lead to greater adiposity and will be stronger for women and those residing in urban areas.

## Methods

### Study design and sampling strategy

Secondary analysis was conducted using data from the Jamaica Health and Lifestyle Survey 2008 (JHLS II), a nationally representative cross-sectional study [9]. Respondents were interviewed using a structured questionnaire on diseases and lifestyle behaviours and anthropometry done.

Full details on the sampling technique for the JHLS II are provided elsewhere [9]. Briefly, Enumeration Districts (EDs) were used as the primary sampling units (PSUs). Enumeration Districts (EDs) are equivalent to a U.S. census tract. They are the smallest geographic unit into which Jamaica is divided to facilitate the collection of census and survey data. A stratified random sample of clusters, the EDs was selected using a probability proportionate to the size of population of each of the 14 parishes to yield a nationally representative sample. Households were systematically selected beginning at a random starting point and based on predetermined sampling intervals to recruit 2914 participants in twelve age-sex categories i.e. male and female 15–24; 25–34; 35–44; 45–54; 55–64; 65–74 years old. Within each cluster, or primary sampling unit (PSU), the sampling interval N was equal to the number of households divided by the sample (agreed to be 30) so that in a PSU with 300 households the sampling interval would be 10. Thus every 10^th^ household, beginning with the Statistical Institute of Jamaica (STATIN) assigned random starting point, was targeted for participant selection. Within each household, a single individual was chosen to participate. The participant from each household was selected by the KISH methodology [9]. Interviewers were required to revisit households where adults were not at home at the time of first contact with the household. A minimum of three visits were made before the household/participant was deemed a refusal [9]. A sample of 2848, 15-74-year-olds was recruited, representing approximately 70% of the Jamaican population and a response rate of 98.3%. Of these, for the secondary analysis, 89% (n = 2529) of participants’ records were geocoded and linked to their EDs. Kreft [40] suggests a ’30/30 rule’ so that researchers should strive for a sample of at least 30 groups with 30 individuals per group. Each of the 101 EDs had an average of 28 individuals, sufficient to power the proposed multilevel analyses, including potential subgroup analyses.

### Measures

#### Individual-level factors

*Outcome variables*. The primary outcomes were mean BMI and mean WC. BMI was calculated as weight divided by height squared (kg/m^2^). Weight was measured using calibrated electronic scales (Tanita® models HD 314 or 2204) to 0.1 kg precision and height measured using a portable stadiometer (Seca®) to 0.1 cm precision. WC measurements were taken at the midpoint between the lower rib margin and iliac crest at relaxed expiration and measured to the nearest 0.1 cm.

*Covariates*. Data collected included age, sex, educational attainment, occupation, urbanicity, PA, perceived community safety and anthropometry, all based on *a priori* theory. Occupation was first categorised using the Jamaica Standardised Occupational Classification codes for 1991 [41] and thereafter regrouped into highly skilled/professional, skilled, unskilled and unemployed/other categories. A geographical area was considered urban if it had a population of 2,000 or more persons and provided a number of amenities and facilities which in Jamaica indicates modern living. The minimum criteria for urbanicity is that the area must have at least one commercial, financial, professional, residential and public service entity [42].

PAL was examined as the frequency and types of PA based on questions on work and leisure-time PAL from a locally developed questionnaire: Low PAL participants engaged in PA that increased breathing and heart rate, lasted at least 20 minutes and was done one or two times weekly; inactivity was defined as persons engaged in PA similar to that for low but less than once per week [43].

Perception of community safety was determined by asking each participant how safe he or she felt to walk in the community. The responses categories were very safe, safe, usually safe, can be dangerous and very dangerous.

#### Household-level factors

Studies done within and outside of Jamaica have identified the economic situation and physical home environment as reliable and valid dimensions of SES. Specific factors include the number of appliances in the home and material possessions [44–46]. The primary analysis of the JHLS II revealed a high non-response rate of 32.65% to questions on income. Therefore, in this study the number of possessions owned was used as a proxy for SES and classification based on the following tertiles: 1^st^ tertile = ≤ 6 items, 2^nd^ tertile = 7–9 items, 3^rd^ tertile = 10–16 items. These included but were not exclusive to owning a radio, telephone, refrigerator, television, computer or car ownership.

#### Neighborhood-level factors

**Fig 1:**
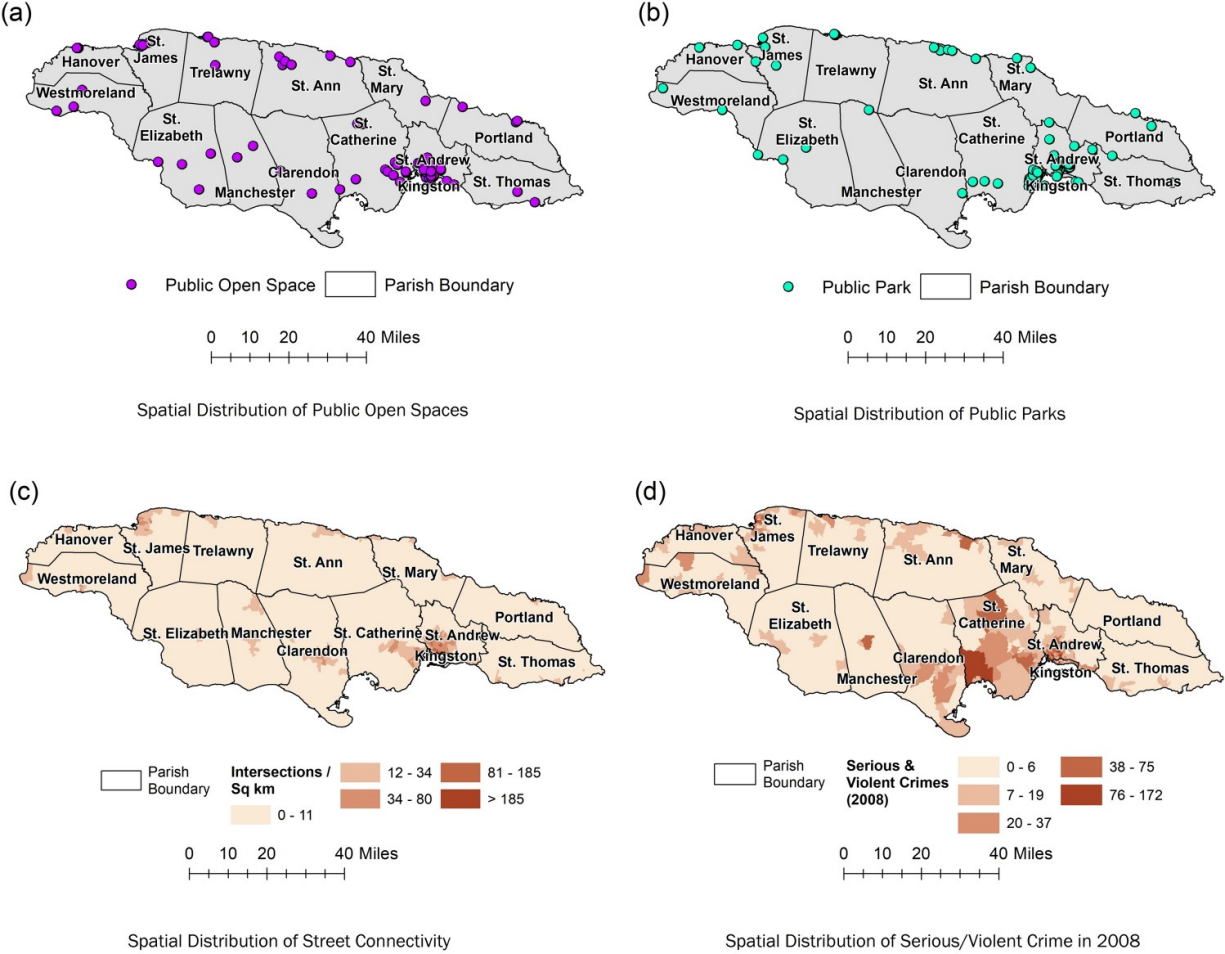
(a) Spatial distribution of public open spaces. (b) Spatial distribution of public parks. (c) Spatial distribution of street connectivity. (d) Spatial distribution of serious/violent crime in 2008.

*Exposure variables*. A total of 2,529 (89%) participants from the JHLS II dataset were geocoded out of the original 2,848 participants. At the end of the mapping process, each respondent was represented spatially in a shapefile (a popular geospatial vector data format for GIS software) and the relevant information (outcomes and risks) were tagged. The mapped data were prepared for analysis at various scales, which included participants by individual location (represented as points of the home address) and by ED, parish and health region scale (represented by a polygon count). Neighbourhood data, defined by an ED, was then joined to each geocoded address to provide the built environmental context. Aggregate level demographic, socio-economic and infrastructural data were also tagged to each geocoded address specific for the ED of residence. Information such as population count and density of public open spaces (green spaces)/playing fields (POS) as well as the subset of public parks, street connectivity, as well as serious and violent crimes were also tagged. These data were obtained from the Mona Geoinformatics Institute (MonaGIS) proprietary JAMNAV database and were collected between 2009 and 2010. The spatial distribution of these neighborhood-level variables is illustrated in Fig 1. The final choices of environment-level measures for investigation were based on a combination of previously derived GIS-based measures [37, 47, 48], documented associations seen with the outcomes of interest and data availability. Further details on the creation of these measurements will be discussed in the next paragraphs.

Public open spaces (green spaces)/playing fields locations were defined as artificially cleared fields developed for the specific purposes of recreation, for example sports, and include both informal and formal areas, that is, areas where entry fees may be charged (Fig 1, Panel a).

Proximity was determined as the straight-line distance in km from each geocoded address to the centroid of the closest public open space using the Spatial Analyst tool in ArcGIS. Two density variables were created including density of public open spaces per km^2^ and the number of public open spaces per 1000 persons in the corresponding ED, was calculated using data in the 2011 census from the Statistical Institute of Jamaica [STATIN] [42].

Public parks locations were defined as areas with no entrance fee and managed by government agencies (Fig 1, Panel b). Proximity and density measures were created similar to that done for public open spaces.

Street connectivity was measured by assessing intersection density (Fig 1, Panel c). Intersections were defined as street network nodes with 3 or more associated street segments. Street connectivity density variables were examined as: i) the number of intersections per km^2^, whereby the number of intersections were counted within a 1 km radius of a participant’s residence, and ii) the number of intersections per km^2^ per ED.

Serious and violent crime rate was assessed by examining serious and violent crimes which were defined as homicides, shootings, robberies aggravated assault and rape for 2008, the year of completion of the JHLS II (Fig 1, Panel d). The data were obtained from the Jamaica Constabulary Force database. Exposure variables included serious and violent crimes density in terms of number /km^2^ for 2008 and number per 1000 persons per ED per year.

*Zero-inflated variables*. The absence of the neighborhood-level factor based on the participant’s geocoded address was indicated by a large proportion of zero values for most density measures (See S1 Table). For example, the variables, Open spaces / km^2^, Open spaces /1000 people/ ED, Public Parks / km^2^, Public Parks / 1000 people/ ED, Intersection density / km^2^ / ED, No. of crimes / km^2^/yr, No. of crimes / 1000 people/ ED/yr had over 10% of zero values.

New indicator variables (dummy variables) were subsequently created and the specific dummy variable included in regression models alongside the original quantitative forms of the respective PA and social environments variables. These dummy variables are also referenced as the zero-inflated form of the density measures.

### Missing data

Of 2848 participants’ records, 355 (12.5%) were excluded from analyses because the addresses lacked sufficient detail to be geocoded. A similar percent of observations for all variables was excluded from analyses due to absence of geocoded respondents. No key deviations were seen when the age/sex population of this geocoded JHLS II secondary analysis subsample was compared with the non-geocoded data.

To assess whether the geocoded responses could be regarded as a simple random sample of the JHLS II study participants and therefore also representative of the Jamaican population, the geocoded and non-geocoded (missing) participants’ records were compared with respect to sex, age, SES categories and the outcome variables of mean BMI and mean WC. Three different approaches were used in the assessment of the associations, namely mixed effect models, regression models accounting for survey design and regression models that ignored survey design. No associations were detected between the geocoded status and non-geocoded records and these other variables.

The missing values for BMI and for mean waist circumference (WC) could be classified as missing completely at random (MCAR), because the probability of their missingness was not dependent on any of the explanatory or outcome variables.

### Statistical analysis

Statistical data analysis provided survey-weighted along with the 95% confidence intervals for the estimates of means (for quantitative variable) and percentages (for qualitative variables) in the sex-specific and total populations. This enabled identification of possible confounders in the relationship between the neighbourhood variables and the adiposity outcomes namely, body mass index (BMI) and waist circumference (WC).

Prior to the investigation of the relationship between the neighbourhood variables and the adiposity outcomes using multilevel linear regression and analysis of covariance models, null models with each of BMI and WC as outcome variables, were used to estimate the intraclass correlation coefficients, which would determine evidence of clustering due to enumeration districts alone or in combination with other levels of variation that define the neighbourhood. These levels were the regional health, the parish, and constituency in which the respondent’s enumeration district was located. The enumeration district and parishes were the bases for calculation of the neighbourhood variable values used in the regression models. The levels used in subsequent hierarchical models that examined association between environment-level variables with the outcomes were those yielding ICC of at least 2% [49]. Thus, while accounting for the effect of clustering on parameter estimates, the random effects linear regression or analysis of covariance models estimated the proportion of variance in mean BMI and WC that can be explained at the higher level, in this case ED.

Initially, unadjusted regression models quantified the relationship between neighbourhood environment variables and the adiposity indices. Next, the unadjusted models were estimated using terms for interaction between either one of sex, urban versus rural area of residence and socioeconomic index defined using number of household possessions. These unadjusted models enabled determination of whether the effect of neighborhood environment on the adiposity indices differed depending on the level of the aforenamed sociodemographic variables. Subsequently, the models with interaction terms were estimated with adjustment for confounders to determine whether any sociodemographic group-specific associations identified were retained in the presence of confounding. The Goodman and Kruskal’s gamma coefficient γ [50] was estimated for each pair of confounder variables to determine whether they were too highly correlated to be used together in the same model. Multi-collinearity between variable pairs was deemed existent if the gamma coefficient exceeded 0.60 or 60%. The Akaike’s Information Criterion (AIC) was the basis for identifying the final model which best explained variation in the outcomes. The residual values from the fully adjusted final models were assessed using the skewness and kurtosis test for normality [51]. If the residuals did not follow the normal distribution, the models were refitted using variable values whose distribution was closer to being a normal distribution, as determined by p-values from the skewness and kurtosis test for normality [51]. In addition, the AIC values for these refitted models, as well as the distribution of the residuals from the models and inference drawn, were compared with the similar results obtained from the models that used the untransformed data.

In recognition of the multiple tests applied to each outcome, the originally estimated *p*-values (*p*) were adjusted using three methods to yield *p* values (*p*′) adjusted for multiple comparisons. The methods applied were the Bonferroni [52, 53], Sidak [53] and Holm [52] methods with such that, respectively, *p*′ = min (1, *np*), *p*′ = 1−(1−*p*)^*n*^ (for *n* equal the number of hypothesis tests) and *p*′ = *min*(1, n(p)×*p*) (for n(*p*) equal the number of *p*-values smaller than *p*).

All analyses were conducted using STATA, versions 12 and 14 (StataCorp LP, College Station, Texas). A *p*-value of < 0.05 was considered significant.

### Ethical considerations and data confidentiality

Ethical approval for the JHLS II was obtained from the University Hospital of the West Indies/University of the West Indies Ethics Committee, and all study participants provided written consent. Confidentiality of all participants and national data were protected within legal limits. Specifically, participants’ addresses were geocoded and merged with the primary dataset based on a unique identifier. The identity of an individual was excluded thereafter, and so data analyses were done on anonymized data. Data were stored by the researcher at the Dept. of Community Health and Psychiatry, University of the West Indies, Jamaica and access limited only to study personnel.

## Results

Descriptive statistics describing the sample population are shown in Table 1. Significantly more men were in the upper SES class based on tertiles of possessions owned (*p*<0.001), and a higher proportions of men vs women (M = 86.29% vs W = 80.38%, *p* = 0.012) perceived their communities as unsafe. Compared to men, women had significantly higher mean BMI and mean WC, and lower levels of physical inactivity (*p*<0.001). When sex differences were assessed for the neighborhood-level environment, we observed that more men lived in areas with a higher proportion of intersection density/km^2^ /ED compared to females (M = 25.61% vs W = 22.02%, *p* = 0.013).

**Table 1:** Total and sex-specific weighted sample characteristics (95% CI) for Jamaicans (JHLS II, 2008).

Variable	Total (n = 2527)	Men (n = 796)	Women (n = 1731)
Individual -level measures			
Mean Age (years)	36.87 (36.54, 37.20)	37.00 (36.33, 37.13)	36.73 (36.64, 37.36)
Urban Residence (%)	53.35 (44.87, 61.64)	53.53 (43.84, 62.28)	53.17 (45.11, 61.74)
Possessions owned (%)***			
≤ 6 items	**37.82 (33.96, 41.68)**	**34.09 (29.03, 39.14)**	**41.51 (37.45, 45.57)**
7–9 items	**30.55 (27.89, 33.20)**	**29.92 (25.99, 33.85)**	**31.17 (28.45, 33.89)**
10–16 items	**31.63 (27.45, 35.82)**	**36.00 (30.68, 41.31)**	**27.32 (22.99, 31.65)**
< High School Education (%)	30.43 (26.96, 33.90)	31.75 (27.17, 36.33)	29.13 (25.69, 32.56)
Occupation (%)***			
Highly skilled/Professional	**45.73 (42.73, 48.72)**	**38.87 (34.41, 43.34)**	**52.54 (49.02, 56.06)**
Skilled	**24.23 (21.29, 27.16)**	**40.33 (35.19, 45.48)**	**8.21 (6.19, 10.23)**
Unskilled	**13.95 (11.33, 16.57)**	**9.75 (6.66, 12.84)**	**18.13 (15.20, 21.06)**
Unemployed/Other	**16.10 (14.12, 18.07)**	**11.05 (8.22, 13.88)**	**21.11 (18.17, 24.06)**
Perception of unsafe community (%)*	**83.32 (79.77, 86.86)**	**86.29 (82.64, 89.94)**	**80.38 (75.63, 85.13)**
Low and Inactive Physical Activity (%)***	**44.55 (41.39, 47.73)**	**27.55 (23.08, 32.02)**	**61.57 (58.10, 65.05)**
Mean BMI^⸸^ [kg/m^2^] ***	**26.64 (26.21, 27.07)**	**24.83(24.28, 25.38)**	**28.40 (27.90, 28.89)**
Mean WC^⸸^ [cm] ***	**85.92 (85.14, 86.69)**	**83.30 (82.23, 84.37)**	**88.46 (87.41, 89.52)**
Neighborhood -level measures^†^ [Mean (95% CI)]			
Open spaces proximity (km)	7.18 (6.12, 8.24)	7.26 (6.19, 8.33)	7.09 (5.91, 8.27)
Open spaces / km^2^	0.06 (0.04, 0.09)	0.06 (0.04, 0.09)	0.07 (0.04, 0.09)
Open spaces /1000 people/ ED	3.00 x10^-8^ (7.90x10^-10^, 5.93 x10^-8^)	4.50 x10^-8^ (-6.37 x10^-9^, 9.65 x10^-8^)	1.52 x10^-8^ (5.60x10^-10^, 2.98 x10^-8^)
Public parks proximity (km)	5.67	5.72	5.62
Public parks / km^2^	0.16	0.16	0.16
Public parks / 1000 people/ ED	0.16	0.18	0.14
Intersection density/ km^2^	27.24	27.26	27.21
Intersection density/ km^2^ / ED*	**23.80**	**25.61**	**22.02**
No. of crimes / km^2^ / year	8.50	8.11	8.88
No. of crimes /1000 people/ ED / yr	4.63	5.10	4.16

^†^ Age-adjusted

* *p*<0.05

** *p*<0.01

*** *p*<0.001 for difference between two means or proportions (men versus women)

CI–Confidence Interval; JHLS II, Jamaica Health and Lifestyle Survey II; BMI—Body Mass Index; ED–Enumeration District; WC–Waist Circumference

Statistically significant estimates are in bold (*p*<0.05)

There was significant clustering of mean BMI (ICC = 4.16%) and mean WC (ICC = 4.34%) at the neighborhood level nested within parishes. While no statistically significant associations were detected between the neighborhood-level environmental exposures and individual-level mean BMI and mean WC in unadjusted models, this may be due to moderating effects. Statistically significant interactions were found between sex, urbanicity and SES and specific geographic variables in their association with each outcome, presented below.

### Mean BMI

The impact of the neighborhood environment, specifically proximity of open spaces, on BMI differed by level of urbanicity (*p*<0.01). A 100% increase in distance away from open spaces, was associated with a 7 kg/m^2^ increase in mean BMI for rural residents (*p* = 0.039), after adjusting for age, sex, and number of possessions. However, this association was attenuated in fully adjusted models (see Table 2).

**Table 2:** Urban-rural-specific unadjusted and adjusted β coefficients (Confidence Intervals in brackets) for the association of open spaces proximity with mean Body Mass Index.

Environment	Mean BMI	
	Urban (n = 1260)	Rural (n = 1233)
	β (95% CI)	β (95% CI)
Open spaces proximity (km)		
Unadjusted	-0.07 (-0.16, 0.01)	0.07 (-2.46x10^-4^, 0.14)
Adjusted^a^	-0.07 (-0.15, 0.01)	**0.08 (0.01, 0.15)** *
Adjusted^b^	-0.06 (-0.14, 0.03)	**0.07 (3.71x10**^**-3**^**, 0.14)** *
Adjusted^c^	-0.04 (-0.12, 0.05)	0.07 (-4.28x10^-3^, 0.14)

BMI–Body Mass Index; CI–Confidence Interval

* *p*<0.05

^a^ age adjusted

^b^ adjusted for age, sex and no. of possessions

^c^ adjusted for age, sex, no. of possessions, occupation, education, physical activity, perception of unsafe community

Statistically significant estimates in bold

We also observed a differential impact of intersection density per km^2^ on BMI by sex (see Table 3). Among men, in fully adjusted models a 10% increase in intersection density was associated with a 0.2 kg/m^2^ increase in mean BMI (*p* = 0.032).

**Table 3:** Sex-specific unadjusted and adjusted β coefficients (Confidence Intervals in brackets) for the association of intersection density/km^2^ with mean Body Mass Index.

Environment	Mean BMI	
	Men (n = 784)	Women (n = 1709)
	β (95% CI)	β (95% CI)
Intersection density/ km^2^		
Unadjusted	**0.02 (7.23x10**^**-4**^**, 0.04)** *	-5.07 x10^-3^ (-0.02, 0.01)
Adjusted^a^	**0.02 (4.48 x10**^**-3**^**, 0.04)** *	-0.01 (-0.02, 0.01)
Adjusted^b^	**0.02 (1.13 x10-**^**3**^**, 0.04)** *	-0.01 (-0.02, 5.62 x10^-3^)
Adjusted^c^	**0.02 (1.96 x10**^**-3**^**, 0.04)** *	-4.48 x10-^3^ (-0.02, 0.01)

BMI–Body Mass Index; CI–Confidence Interval

* *p*<0.05

^a^ age adjusted

^b^ adjusted for age and no. of possessions

^c^ adjusted for age, no. of possessions, urban, occupation, education, physical activity, perception of unsafe community

Statistically significant estimates in bold

A differential impact of public parks proximity and absence of crimes per 1000 people per ED per year on mean BMI was also seen by SES. Among persons within the 2^nd^ SES tertile (middle-class), significant positive relationships were seen in all models for the relationship between public parks proximity with mean BMI except in the final model (see Table 4). In the models adjusted for age and sex, a 10% increase in the distance from a public park was associated with a 1.1 kg/m^2^ increase in mean BMI (*p* = 0.015). When the association between absence of crimes/1000 people/ED per year and mean BMI was examined the association was only statistically significant in unadjusted models in the second tertile–middle class (*p* = 0.0496).

**Table 4:** SES-specific unadjusted and adjusted β coefficients (Confidence Intervals in brackets) for the association of public parks proximity and number of crimes/1000 people/ ED/yr with mean Body Mass Index.

Environments	Mean BMI		
	1^st^ SES tertile (n = 1064)	2^nd^ SES tertile (n = 752)	3^rd^ SES tertile (n = 667)
	β (95% CI)	β (95% CI)	β (95% CI)
Public Parks proximity			
Unadjusted	-0.03 (-0.11, 0.45)	**0.12 (0.02, 0.21)** *	0.02 (-0.11, 0.14)
Adjusted^a^	-0.03 (-0.11, 0.05)	**0.10 (0.01, 0.20)** *	0.02 (-0.10, 0.14)
Adjusted^b^	-0.02 (-0.10, 0.05)	**0.11 (0.02, 0.20)** *	0.01 (-0.11, 0.12)
Adjusted^c^	0.97 (0.94, 1.02)	1.02 (9.97 x10^-1^, 1.10)	1.05 (0.99, 1.12)
No. of crimes/1000 people/ ED/yr^**ǂ**^			
Unadjusted	-0.39 (-1.33, 0.55)	**1.17 (2.12 x 10**^**−3**^**, 2.33)** *	-0.44 (-1.74, 0.87)
Adjusted^a^	-0.22 (-1.14, 0.70)	1.05 (-0.09, 2.19)	-0.26 (-1.54, 1.01)
Adjusted^b^	-0.15(-1.03, 0.74)	1.00 (-0.09, 2.09)	0.18 (-1.03, 1.40)
Adjusted^c^	-0.25 (-1.23, 0.73)	1.17 (0.03, 2.36)	-0.25 (-1.05, 1.55)

BMI–Body Mass Index; CI–Confidence Interval; ED–Enumeration District; SES–Socioeconomic Status

^ǂ^ Dummy variable for zero inflated predictor

* *p*<0.05

^a^ age adjusted

^b^ adjusted for age and sex

^c^ adjusted for age, sex, urban, occupation, education, physical activity, perception of unsafe community

Statistically significant estimates in bold

### Mean WC

Effect modification by urbanicity was also observed in the association between the number of crimes/km^2^/yr and mean WC (*p* = 0.045). Table 5 shows that a one-unit increase in number of crimes/ km^2^/yr. was associated with at least a 0.08 cm increase in mean WC in all models for urban residents.

**Table 5:** Urban-rural-specific unadjusted and adjusted β coefficients (Confidence Intervals in brackets) for the association of no. of crimes / km^2^/yr with mean waist circumference.

Environment	Mean WC	
	Urban (n = 1260)	Rural (n = 1233)
	β (95% CI)	β (95% CI)
No. of crimes / km^2^/yr		
Unadjusted	**0.08 (0.01, 0.15)** *	-0.33 (-0.07, 0.06)
Adjusted^a^	**0.09 (0.03, 0.15)** **	-0.27 (-0.63, 0.10)
Adjusted^b^	**0.08 (0.01, 0.14)** *	-0.22 (-0.58, 0.14)
Adjusted^c^	**0.09 (0.03, 0.16)** **	-0.26 (-0.62, 0.10)

CI–Confidence Interval; WC–Waist Circumference

* *p*<0.05

** *p*<0.01

^a^ age adjusted

^b^ adjusted for age, sex and no. of possessions

^c^ adjusted for age, sex, no. of possessions, occupation, education, physical activity, perception of unsafe community

Statistically significant estimates in bold

Effect modification by SES was also observed in associations of i) public parks proximity (*p* = 0.011) and ii) intersection density per km^2^ per ED (*p* = 0.011) and mean WC. All models that examined the association of public parks proximity with mean WC were statistically significant in the 2^nd^ SES tertile (Table 6). Specifically, among the middle class a 10% change in proximity to public parks was associated with a 3 cm increase in mean WC in models adjusted for all covariates (*p*<0.01). Table 6 also shows that a 100% increase in intersection density per km^2^ / ED was associated with a 4 cm decrease in mean WC only in the unadjusted models for the 3^rd^ SES tertile–upper class (*p* = 0.04).

**Table 6:** SES-specific unadjusted and adjusted β coefficients (Confidence Intervals in brackets) for the association of public parks proximity and intersection density/ km^2^ / ED with mean Waist Circumference.

Environments	Mean WC		
	1^st^ SES tertile (n = 1064)	2^nd^ SES tertile (n = 752)	3^rd^ SES tertile (n = 667)
	β (95% CI)	β (95% CI)	β (95% CI)
Public Parks proximity(km)			
Unadjusted	-0.04 (-0.22, 0.14)	**0.26 (0.05, 0.48)** *	0.12 (-0.15, 0.39)
Adjusted^a^	-0.01 (-0.18, 0.15)	**0.24 (0.04, 0.45)** *	0.15 (-0.11, 0.41)
Adjusted^b^	-0.01 (-0.17, 0.15)	**0.25 (0.05, 0.45)** *	0.12 (-0.14, 0.37)
Adjusted^c^	0.36 (-0.15, 0.22)	**0.30 (0.08, 0.53)** **	0.27 (-0.02, 0.57)
Intersection density/ km^2^ / ED			
Unadjusted	0.02 (-0.01, 0.05)	-0.01 (-0.05, 0.03)	**-0.04 (-0.08, -1.69x10**^**-3**^**)** *
Adjusted^a^	0.02 (-0.01, 0.04)	-0.01 (-0.05, 0.03)	-0.03 (-0.07, 3.09 x 10^−3^)
Adjusted^b^	0.02 (-0.01, 0.04)	-3.67 x 10^−3^ (-0.04, 0.03)	0.03 (-0.06, 0.01)
Adjusted^c^	8.53 x10^-3^ (-0.02, 0.04)	-0.02 (-0.05, 0.02)	-0.02 (-0.06, 0.01)

CI–Confidence Interval; ED–Enumeration District; SES–Socioeconomic Status; WC–Waist Circumference

^**ǂ**^ Dummy variable for zero inflated predictor

* *p*<0.05

** *p*<0.01

^a^ age adjusted

^b^ adjusted for age and sex

^c^ adjusted for age, sex, urban, occupation, education, physical activity, perception of unsafe community

Statistically significant estimates in bold

### Assessment of model adequacy

The fully adjusted final models as determined by the minimum values of AIC had outcome variable values that did not follow the normal distribution, nor did the residual values from these models. The models were refitted using the square root, reciprocal of the square root and logarithm to the base e transformations of the outcome variables. Each transformation yielded a model that was a substantial improvement on the model produced by the original (untransformed) data as the reduction in AIC was larger than 10 [54], although not all the residuals from the refitted models had a normal distribution. The conclusions drawn from the use of transformed data remained the same as for models that used untransformed data.

### Multiple testing

Multiple formal group comparisons were tested in models fitted when the effects of the geographic variables on the outcomes in the total sample were assessed. The p-values corrected for multiple comparisons revealed that the interaction terms that were previously statistically significant remained so only when the Holm method of correction was applied (See S1 Data).

## Discussion

This study is the first to examine the impact of neighborhood physical and social environments on adiposity in a small Caribbean island. There was significant clustering across neighborhoods for mean BMI (ICC = 4.16%) and mean WC (ICC = 4.42%). Concerning the physical environments for PA, we observed that: a) a further distance away from open spaces was associated with increased mean BMI in rural residents; b) a further distance away from public parks was associated with increased mean BMI and mean WC among middle class residents; c) increased intersection density/km^2^/ED was statistically significantly associated with lower mean WC among middle class residents; d) contrary to our hypothesis, greater street connectivity as measured by intersection density/km^2^ was associated with increased mean BMI in men.

In terms of the social environment, the number of crimes per km^2^ per year was associated with increased mean WC among urban residents. No statistically significant sex differences were detected as hypothesized between this social environment variable and mean WC.

Results corroborate other studies internationally and locally, which have reported high neighbourhood clustering for obesity-related outcomes [55, 56], including mean BMI and mean WC, indicating the importance of environmental influences in their variance and supporting our findings.

Being closer to open spaces/public parks and higher street connectivity are indicators of neighbourhood walkability [25, 57], which may decrease obesity-related outcomes [33]. Our findings corroborate previous research, but with important and unexpected differences across geography and SES in some adjusted models. We noted a difference with directionality of these associations whereby among rural residents, further distance away from open spaces increased mean BMI, and the opposite was so for urban. It is unclear what is influencing this disparity, but it is possible that the nature of the open spaces may differ for each setting whereby greater spaces in urban areas may be linked to greater levels of physical activity if that space is more like a park. This urban/rural difference warrants further exploration, particularly on the accessibility and attractiveness of the open spaces which are associated with use for PA [26], as well as the utility or type of that space, and may possibly vary between both settings.

Results also suggest that proximity to public parks and intersection density may play a role in obesity-related outcomes for the middle class, although models examining these physical environments did not adjust for diet, based on the hypothesized mechanistic pathway. It is possible that if poor diet had been operationalized differently and adjusted for in the final regression models, its role as a statistically significant confounder might have been demonstrated. In reviewing the related literature some reported associations with obesity among the middle class was found in Bangladesh and China [58, 59]. In addition, we used number of possessions as the indicator of SES. However other studies have suggested that education is the most robust and stable SES indicator [60].

Contrary to our hypothesis, among men, greater street connectivity as measured by intersection density/km^2^ was associated with increased mean BMI. This surprising finding needs further exploration as it may be related to the type of PA that Jamaican men engage in on streets, and/or time spent in vehicular transport, such as cars. For example, in Jamaica most areas with high street connectivity areas are coterminous with high population density areas which are often in lower SES neighborhoods. Additionally, is not unusual to see males engaged in soccer on the streets of these communities. In the USA, Oakes and colleagues found that high population density areas were more associated with increased odds of travel related walking [61] but the researchers did not report on sex differences. No association of street connectivity with measures of adiposity in women was found in our study. Finally, another possible reason for the association seen in men, is that neighborhoods with higher intersection density may have higher crime rates and/or greater availability of fast food/prepared foods/street vendors, which could in turn account for the higher BMIs observed in men in these neighborhoods.

In keeping with the study hypothesis on the crime environment, the number of crimes per km^2^ per year was associated with increased mean WC among urban residents. We used objectively measured crime data; however, the literature has revealed inconsistencies in the association between perceived versus objectively measured neighborhood crime levels with either PAL or obesity-related measures as the dependent variables. For example, perceptions of crime related safety have been found to constrain PALs among women and older adults where actual levels of crime are lower than perceived, suggesting that sometimes perceptions rather than objective measures may have a more powerful effect on behavior [62]. No statistically significant sex differences were found as hypothesized.

### Strengths and limitations of the study

Despite many strengths of the study, including a nationally representative sample of Jamaicans and one of the first studies to examine neighborhood physical and social contexts, there are also limitations. These include the inability to make causal inferences given the cross-sectional design. Also, the inclusion of self-reported data and the fact that the reliability and validity of the area-level environmental variables were untested in Jamaica may have introduced information bias. The design of the JHLS II study using stratified, random, two-stage cluster sampling precluded the use of statistical analysis to assess spatial autocorrelation. This would have allowed the assessment of whether there is clustering of the various measures at a global level e.g. Moran’s I statistic or local level e.g. using the local indicator of spatial autocorrelation (LISA).

Additional limitations include the possible inadequacy in using EDs to represent a Jamaican neighborhood given their heterogeneity geographically in size, composition and context and the cut-offs used to signal increased CVD risk, which were mainly derived from European populations with some studies on persons of African descent [63] suggesting the use of higher threshold cut-points for increased CVD risk—these may have resulted in over or under estimation of the effects seen. Furthermore, there was temporal mismatch of the data collected from individual JHLS II participants with that for the physical and crime environment-level variables. These neighborhood-level variables were collected by MonaGIS during 2009–2010, subsequent to the end of data collection for the JHLS II in 2008. This may have biased the results as individual exposures may have varied after the survey period, although the both environments are believed to have been relatively stable over that time and the findings may still be relevant. We also did not assess length of streets, network distance, streets with sidewalks and access to other features such as gyms that capture other nuances of the built environment and maybe associated with obesity, which may have also led to over or underestimation of the effects seen. Assessing the appropriateness and validity of environmental measures will be an important area for future research. Additionally, the data sources did not allow for control for important attitudinal factors that influence lifestyle behaviours such as neighborhood self-selection, social support and social capital. Future qualitative research is needed that focuses on understanding the contribution of these attitudinal factors to obesity outcomes.

The PAL measures used were locally developed and not validated. Although a number of items from the International Physical Activity Questionnaire (IPAQ) Short Last 7 days Self-Administered Format for Use with Young And Middle-Aged Adults (15–69 years) format were included [64], easy comparison with studies in other contexts many not be possible. The variable definition used for PAL was also subject to measurement error which may have perturbed the associations revealed. Further, other covariates, including dietary behaviours, may partially explain the study results, but were not explored in these analyses, based on the hypotheses we wanted to test. We also saw associations at the ED level but recognize the neighborhood influence could be different at another level. Although different measures of exposure, namely proximity and density at the ED level were used, there is the potential for exposure misclassification due to the modifiable areal unit problem (MAUP). MAUP arises from aggregating point-based measures of spatial phenomena to arbitrarily defined geographic areas and the use of administrative boundaries as a proxy for neighborhood. Although EDs are the smallest unit of aggregation in Jamaica to facilitate the collection of census and survey data, they provide only a rough measure of neighborhood context [65].

Lastly, although the statistically significant associations were retained when the p-values were compared with the Holm corrected significance level, this was not the case when compared with the Sidak or Bonferroni corrected significance levels. This could be evidence that a larger sample size would be required to assess these group-specific associations. Both the Sidak and Bonferroni correction, however, are more conservative tests [52, 66, 67]. The use of adjustments for multiple comparisons is also controversial and even discouraged by some authors [66] especially in exploratory studies such as this one. Alternately, resampling techniques such as cross-validation, the jackknife, and the bootstrap could be utilized in subsequent analyses. These resampling methods may identify the true nature of the discrepancy between the true and apparent errors in estimation as obtained for this body of work. The results, nevertheless, suggest directions in the nature of the associations that are worth exploring in subsequent analyses, forming the basis for more in-depth examination of the role of the neighbourhood environment in the development of adiposity with sociodemographic subgroups as the relationship appears to vary with sociodemographic level. These results highlight the important role that both physical and social (crime) environments may play in obesity-related outcomes in the Jamaican context. We recognize that a number of years have ensued since this study was undertaken, however we believe the public health relevance of our findings may still be of importance given the physical environment has remained relatively unchanged and homicide rates rank within the top ten countries globally [68].

### Public health implications

All factors considered, location of public parks, street connectivity, and crime rate reduction are amenable to structural interventions and policy changes. Given the limited resources available for government programmes, the need for more cost-effective approaches in CNCD prevention and control is now an imperative. National strategic and operational plans crafted for prevention and control of CNCDs, for which obesity is a key risk factor, should consider placing greater emphasis on policies, programmes and interventions that are focused on the neighbourhood-level effects and not mainly on individual-level determinants. Greater intersectoral collaboration is needed in decreasing the influence of the physical and social obesogenic environments examined. For example, findings from this study suggest that greater value needs to be placed on the proximity of neighbourhoods to open spaces/public parks. National standards should be developed or revised as to the minimal requirements necessary to facilitate use of these spaces to increase PALs. Community perspectives and participation should be an integral part of any decision–making process. Additionally, evaluation research is needed to assess whether current public investment in existing public parks is yielding health benefits, such as increased PAL. Given the finding that increased intersection density in upper class communities, is associated with decreased WC, urban planners and housing developers should be apprised of this important association as they plan the design of new residential developments for the middle and lower classes. Increased walking should also be encouraged in business districts with high intersection density by applying interventions that discourage driving in these areas, as is done in some large cities in developed countries, for example, London in the United Kingdom.

## Supporting information

S1 Table Proportion of zero-valued observations for geographic variables. (DOCX)

S1 Data Results_corrected p-values Bonferroni, Holm and Sidak multiple comparison tests. (XLSX)

S2 Data (XLSX)
