# Supplementary material for: Associations of neighborhood physical and crime environments with obesity-related outcomes in Jamaica
Source: PLoS One. 2021 Apr 5;16(4):e0249619. doi: 10.1371/journal.pone.0249619 (PMC8021199; doi:10.1371/journal.pone.0249619)
Supplement: S1 Table — (DOCX) [file pone.0249619.s001.docx]

**S1 Table. Proportion of zero-valued observations**

| **Obesogenic Environments** |  | **Frequency (%)**  **N=2527** |
| --- | --- | --- |
| ***Physical*** |  |  |
| Open spaces proximity (km)  Open spaces / km^2^  Open spaces /1000 people/ ED  Public Parks proximity (km)  Public Parks / km^2^  Public Parks / 1000 people/ ED  Intersection density / km^2^  Intersection density / km^2^ / ED |  | 0  17.85  98.54  0  72.69  96.32  8.90  13.02 |
| ***Social*** |  |  |
| No. of crimes / km^2^/yr  No. of crimes / 1000 people/ ED/yr |  | 29.24  47.92 |

ED – Enumeration District
